# Characterization and Agreement Between Application of Mobile Ecological Momentary Assessment (mEMA) and Accelerometry in the Identification of Prevalence of Sedentary Behavior (SB) in Young Adults

**DOI:** 10.3389/fpsyg.2019.00720

**Published:** 2019-04-09

**Authors:** Catiana Leila Possamai Romanzini, Marcelo Romanzini, Cynthia Correa Lopes Barbosa, Mariana Biagi Batista, Gabriela Blasquez Shigaki, Enio Ricardo Vaz Ronque

**Affiliations:** ^1^Department of Physical Education, Londrina State University, Londrina, Brazil; ^2^Academic Department of Humanities, Federal Technological University of Paraná, Apucarana, Brazil; ^3^Physical Education Course, Federal University of Mato Grosso do Sul, Corumbá, Brazil; ^4^Department of Physical Education, Rio Preto University Center and “Paulista” University, São José do Rio Preto, Brazil

**Keywords:** accelerometers, mobile phones, sedentary lifestyle, adults, mobile applications, ecological momentary assessment

## Abstract

New technologies able to identify the sedentary behavior (SB), such as the Mobile Ecological Momentary Assessment (mEMA) still need to be investigated. The aim of this study was to describe SB in the physical, social, and environmental contexts and verify the agreement between the mEMA and accelerometry in the identification of SB in young adults. During 7 days, 123 young adults used concomitant mEMA and Actigraph wGT3xBT accelerometer. Data of 2262 mEMA prompts and respective count values in each minute (5 min previous to prompt) were included in the analyses. Descriptive and comparative statistics were used in analyses using the SPSS 20.0 software. The physical context (PC) at home was the highest occurrence of SB (46.3%) and the main activity was “watching TV/movies” (29.7%). The main social context (SC) related to SB was “staying alone” (49.6%). The main assertions related to the participants’ environmental context (EC) were: “I appreciate the comfort of electronic devices when I am at home” (86.2%). mEMA identified the presence of SB in 78.1% of prompts, while accelerometry identified 70.9% (PABAK = 0.42). High values for the presence of SB were observed (sensitivity = 84%) and lower in the absence of SB (specificity = 38%). The study demonstrates the viability of mEMA use to obtain information about the occurrence of SB in contextual factors and good sensitivity to identify the presence of SB in young adults. The combined use of these methods is suggested in future studies about SB in young adults.

## Introduction

Sedentary behavior (SB) is defined as any activity performed during “awake” time with low energy expenditure (equal to or below 1.5 metabolic equivalents – MET’s) in a sitting or reclining position (Sedentary Behaviour Research Network, 2012; Tremblay et al., 2017). Studies have shown that this behavior is highly prevalent among adults, and most of the time awake (62%) is spent on this type of activity (Hansen et al., 2012) and that the mean percentage of SB, weighted by the total time of accelerometer use per day in a sample of the National Sample of the United States was from 35 to 82.3% (Evenson et al., 2015).

These high SB prevalence rates demonstrated in epidemiological studies indicates that SB is associated with all-cause and cardiovascular morbidity and mortality in adults (Young et al., 2016). Additionally, a recent study based on data for more than 1 million participants in 19 studies has shown that daily sedentary time is log-linearly associated with increased risk of all-cause mortality in adults and suggest that the ideal situation is to spend less than 9 h per day when referring to all-cause mortality (Ku et al., 2018).

Methodologies for assessing SB include self-report and objective measurement, and each provides distinct information and has different limitations (Gibbs et al., 2015). While self-report depends on participant’s ability to remember past activities, objective measurement is not able to identify the different contexts where behavior occurs (Healy et al., 2011; Atkin et al., 2012). Recently, researchers have indicated the need for the development and validation of novel devices capable of assessing posture and standardization of research practices for SB assessment by accelerometry (Gibbs et al., 2015). Ecological Momentary Assessment (EMA) (Shiffman et al., 2008; Atkin et al., 2012) using mobile phones, such as the Mobile Ecological Momentary Assessment (mEMA) application (Runyan and Steinke, 2015) has potential to capture information about the context in which the behavior occurs and the type of activity being performed (Loveday et al., 2016).

A recent study examined the association between EMA records for TV, video, and game use with objective measures of sedentary time in children measured by accelerometer during a 2-h observation window found that EMA records are highly related to the accelerometer measurement. Therefore, understanding the relationship between EMA and accelerometry can optimize future studies aimed at assessing activities and health outcomes (Zink et al., 2018). In adults, few studies have been conducted to verify the validity or agreement between objective SB methods and EMA, and in addition different criteria have been used in data interpretation (Dunton et al., 2012; Liao et al., 2014; Bruening et al., 2016; Knell et al., 2017). The use of EMA in combination with objective measurements (Dunton et al., 2012) can provide information about the presence or absence of SB in different contexts. Until then, studies have been aimed at verifying the relationship between total SB time and health outcomes, but with the possibility for researchers to identify the time spent in SB in different contexts, these new relationships with health outcomes still need to be investigated (Busschaert et al., 2015).

The evidence that SB is associated to health outcomes is still limited because these relationships are almost exclusively measured by self-report (Gibbs et al., 2015) and little explored by methods like EMA, which can obtain information about the context of SB. Therefore, the aim of this study was to describe SB in the physical, social, and environmental contexts and verify the agreement between the application of mEMA and accelerometry in the identification of SB in young adults.

## Materials and Methods

### Participants

This study included 126 young adults who met the following: (a) age between 18 and 25 years; (b) agree to use accelerometer and a mobile phone application simultaneously; (c) have the Android (above 4.2) or mac IOS (above 8.0) system in the mobile phone; and (d) have almost one valid day of accelerometer use. This study was approved by the Londrina State University Human Research Ethics Committee, with number 1.340.735 of November 27, 2015. All young adults signed a written informed consent from to participate in this study.

### Measurement of the SB Context

**FIGURE 1:**
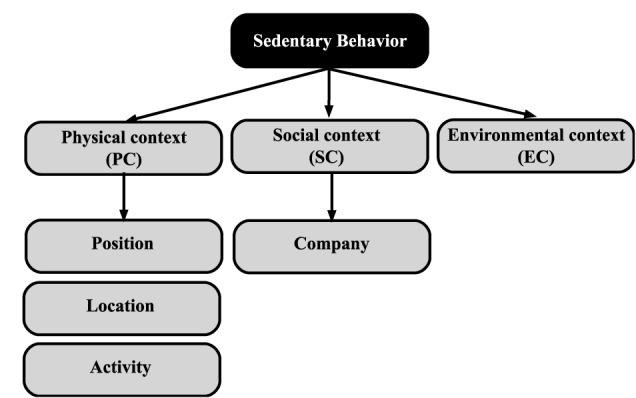
Sedentary behavior (SB) and contextual factors.

One questionnaire about SB and contextual factors was elaborated and inserted in mEMA (Ilumivu.Inc.) that was installed in each mobile phone of participants. The mEMA application was programmed to trigger random alarms during seven consecutive days each 120 min. In weekdays, eight trigger alarms and in weekends, nine trigger alarms were programmed. Upon receiving a phone signal, participants were instructed to stop their current activity and complete a short electronic mEMA question sequence about SB: physical context (PC), social context (SC), and environmental context (EC) (Figure 1).

**FIGURE 2:**
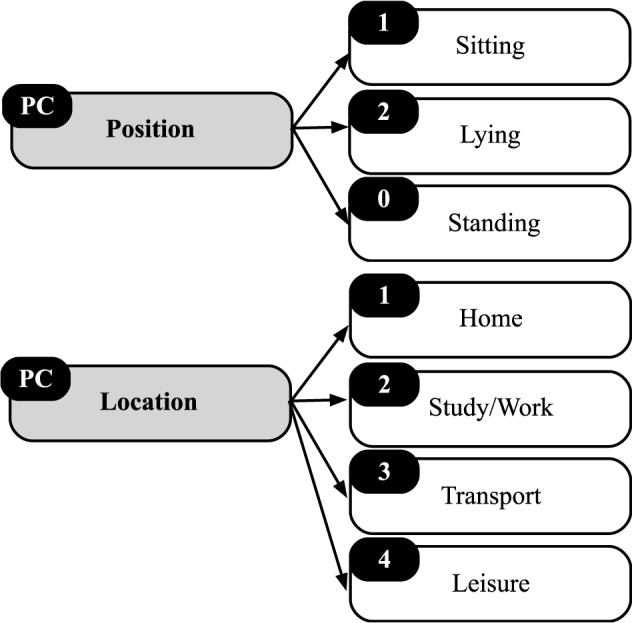
Position and location categories in physical context (PC).

In PC, there are three categories: position, location, and activity. Regarding position participants were asked: “In this exact moment before answering these questions, you were…,” and regarding location: “Where are you doing this activity?” (Figure 2).

**FIGURE 3:**
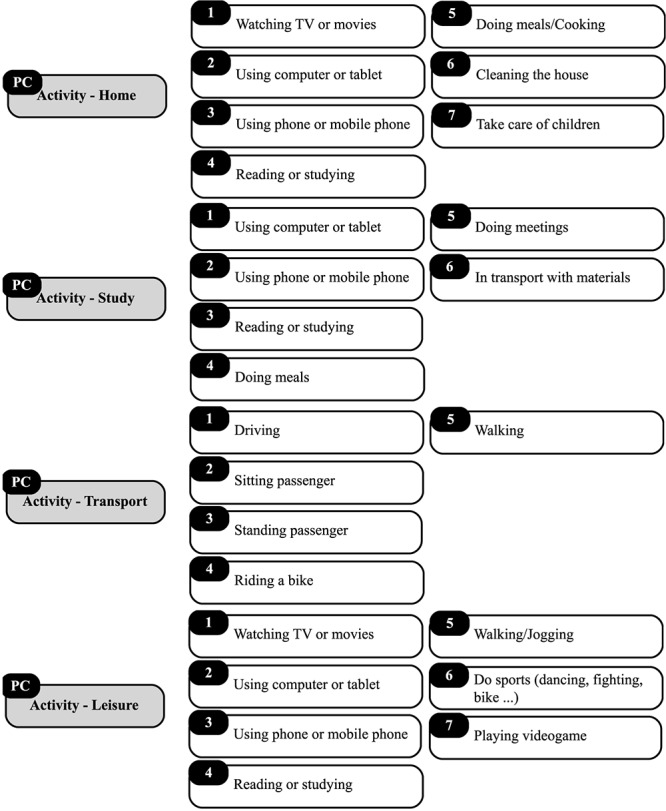
Activity category in PC.

Regarding activity, participants were asked: “In which type of activity were you involved at…? (Figure 3). This question was dependent on the category that the participant reported in item Location, which can be as follows: Home, Study/Work, Transport, and Leisure.

**FIGURE 4:**
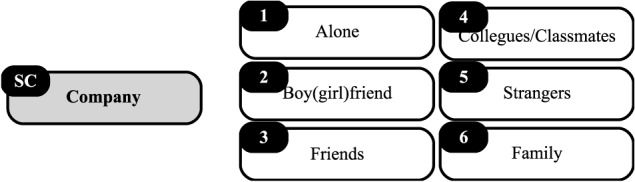
Social context (SC) items.

In the SC, participants answered “Who were with during this activity?” (Figure 4).

**FIGURE 5:**
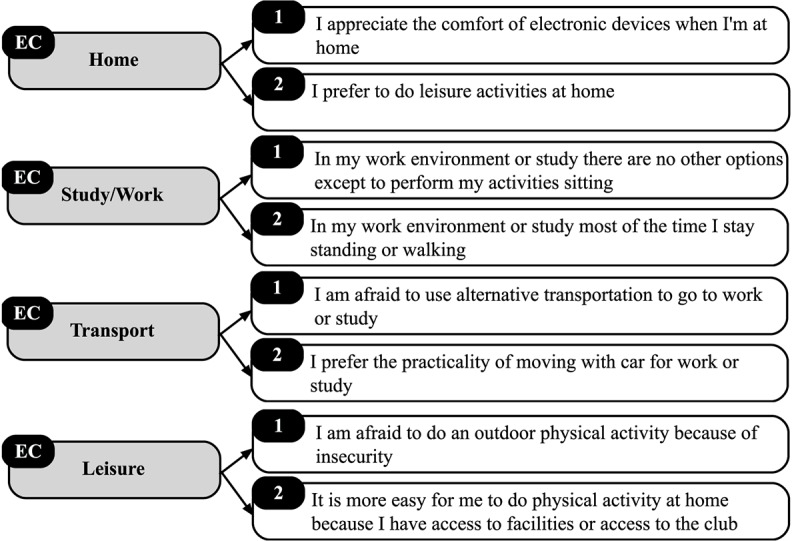
Environmental context (EC) items.

Finally, in EC, the participants should respond “Which of these alternatives below are true for you at…?” (Figure 5). Questions related to EC were requested in a single moment from participants, usually at the beginning of the study when the mEMA was installed. For these questions, some options on the perceived environment were listed for the participant to indicate which of them applied to his/her everyday life. These options were based on the ecological model of four SB contexts (Owen et al., 2011), thus, each participant only made a record of this item choosing the most adequate answer.

If a signal occurred during an incompatible activity (i.e., driving, sleeping or bathing), participants were instructed to ignore it. If no entry was made, the phone emitted up to three reminding signals at 7-min intervals. In addition, participants were instructed to send an upload in mEMA data at the end of each day.

### Objective SB Measure

All participants were monitored by an ActiGraph wGT3x-BT (ActiGraph Pensacola, Pensacola, FL, United States) accelerometer, which was placed on the right hip for 7 days consecutively. They were advised to remove the accelerometers during night time sleep and during any water-based activities. The criteria for reducing the accelerometer data were made in Actilife software version 6.13.3 and defined as proposed by Troiano et al. (2007). It was determined that the time without using the accelerometer referred to a period of at least 60 min of consecutive zeros, with tolerance of one to 2 min of counts between 0 and 100 and a valid day considered with at least 8 h of accelerometer use. At least one valid day of the *x*-axis (vertical) of the accelerometer was considered for the analysis of data.

### Data Analysis

Data from 126 participants generated the possibility of analysis of 3,463 mEMA prompts. Following the exclusion criteria, 660 mEMA prompts were excluded due to the absence of concomitant records in time (mEMA and accelerometer); 19 mEMA prompts presented inconsistencies between the position adopted and the type of activity (e.g., standing – seated passenger) and; 522 mEMA prompts were excluded because it was not possible to classify as presence or absence of the SB outcome (activities classified by the participant as “other” within each of the four contexts). Following the process of excluding these prompts, three participants were no longer represented. Thus, data from 123 participants concerning 2262 mEMA records were used in analysis.

All mEMA data were exported to an excel spreadsheet and the response numbers were encoded. For the classification into presence or absence of SB for the activities reported in the mEMA application, the SB definition and the Compendium of Physical Activities (Ainsworth et al., 1993, 2000, 2011) were used to verify MET’s. For example, the mEMA activity indicated the PC, sitting at home, watching TV or movies, was classified as presence of SB, mainly due to the adoption of the sitting position. On the other hand, the standing position implied in the classification of the behavior as absence of SB.

Accelerometer data were also exported to an excel spreadsheet. Each mEMA prompt was matched of each previous 5 min of the vertical axis of accelerometer. Each minute were classified as presence of SB (<100 counts/min) or absence of SB (≥100 counts/min) (Freedson et al., 1998). Thus, if three or more minutes were classified as presence of SB, this correspondence was classified as presence of SB.

Analyses were processed by SPSS 20.0 for Windows. Descriptive statistics by mean and standard deviation were used in the sample characterization for continuous variables, as well as the proportion for the categorical variables. The significance level was 5%. The agreement between the classification of the presence (1) and absence of SB (0) in mEMA prompts and the accelerometer measurement were tested by prevalence-adjusted bias-adjusted Kappa (PABAK), and by sensitivity and specificity analyses.

## Results

### Occurrence of SB in Physical, Social, and Environmental Contexts

More than half of the participants had response rates of mEMA prompts equivalent to values above 60%. SB was reported through the mEMA application in 78.1% of records. The main place of SB occurrence was at “home” (46.3%), followed by “work” (32.7%). The main activities reported by participants when at “home” were: “watching TV/movies” (29.7%) and “making meals” (18.8%). At the “work” participants reported “using tablet/computer” (46.3%), followed by “reading/studying” (20.8%). At transport participants reported “driving” (58.5%), followed by “sitting passenger” (23.5%), and at leisure “walking” (17.6%) followed by “resistant training” (16.0%). The main SC related to SB was being “alone” (49.6%).

The main assertions related to the participants’ EC at home, study/work, transport and leisure, respectively were: “I appreciate the comfort of electronic devices when I’m at home” (86.2%), “In my work environment or study there are no other options except to perform my activities sitting” (69.9%), “I prefer the practicality of moving with car for work or study” (72.4%) and “It is more easy for me to do physical activity at home because I have access to facilities or access to the club” (20.3%). For this context, participants could choose more than one assertion.

### Agreement Between Presence and Absence of SB for mEMA and Accelerometry

For the minute-by-minute analysis on the concordance between mEMA and accelerometry (5 min previous to mEMA prompts), 11310 records were counted (2262 mEMA prompts multiplied by 5 min). While mEMA application identified presence of SB in 78.1%, accelerometry identified 70.9% (Table 1). Correspondence rates for the mEMA records for presence of SB were 76.0% (6785/8830) and for the absence of SB, correspondence rates were 50.0% (1241/2480).

**Table 1:** Absolute and relative frequency (*n* and %) of presence and absence of SB using Mobile Ecological Momentary Assessment (mEMA) and accelerometry.

mEMA	Accelerometry		Total *n* (%)
	Yes *n* (%)	No *n* (%)	
Yes	6785 (60.0%)	2045 (18.1%)	8830 (78.1%)
No	1239 (10.9%)	1241 (11.0%)	2480 (21.9%)
Total	8024 (70.9%)	3286 (29.1%)	11310 (100.0%)


**mEMA*, application of ecological momentary assessment. PABAK = 0.42. Sensitivity = 84%; Specificity = 38%. (ROC curve area = 0.61, CI = 0.60–0.62, *p* < 0.001).*

The PABAK agreement coefficient was 0.42 and indicated moderate concordance (Landis and Koch, 1977) among records analyzed. The sensitivity and specificity analysis between the mEMA and accelerometry for the presence of SB was tested and showed high sensitivity value (84%). The specificity value was 38% to detect the absence of SB (ROC curve area = 0.61, CI = 0.60–0.62, *p* < 0.001).

## Discussion

The present study was used an innovative method to measure SB in 123 young adults in an attempt to obtain information about these phenomena in different contexts (physical, social, and environmental). The mEMA application presented moderated agreement with accelerometry and can be used to identify the presence of SB in 78.1% of records.

Despite using a different strategy for analyzing EMA data with accelerometer data (± 15 min), lower SB rates (39.6 and 44.4%) were found in two other studies, respectively (Dunton et al., 2012; Liao et al., 2014). Home was the most cited PC in study carried out by Liao et al. (2014), which also used EMA as a method to identify SB in adults (76.0%). SB proportion (41%) similar to that of the present study (50%) was observed in the SC (being “alone”) (Liao et al., 2014). Questions about EC (e.g., presence of nearby trees, and traffic intensity), were requested by EMA application (Liao et al., 2014), however, this information was only considered if the participant was practicing physical activity and not in SB.

One study that investigated the agreement between information from the EMA application and accelerometry data analyzed 694 records from 41 participants (Bruening et al., 2016). Although this study used different criteria in the analysis of records (e.g., mean of counts/min of the vertical axis of the accelerometer), different data agreement parameters of these studies could be compared to those of the present study. Correspondence rates of 76.0% for presence of SB were close to the values of the study above (60.3%). Sensitivity values of 84% and specificity of 38% to detect the presence and absence of SB, respectively, were also quite close to those of the study above (sensitivity of 91% and specificity of 30%, respectively) (Bruening et al., 2016).

Another recent study on physical activity validation with EMA using secondary data from the Socioeconomic Status and Behavioral Cancer Risk Factors Study (PATCH), showed that EMA in the cell phone was a practical alternative to measure physical activity in contexts of everyday life (Knell et al., 2017). For the SB measurement, the daily average estimated for the total number of days that participants used the accelerometer was used (less than 4 days of use). In relation to SB, this study showed that the EMA and the SB time determined by the accelerometer were correlated (*r* = 0.16; *p* < 0.05) and that there was no statistical significance in the agreement between SB time determined by the accelerometer and by self-reported measures (Knell et al., 2017).

In similar study conducted with children, it was observed that EMA-reported TV, videos, or video games was associated with a greater accelerometer-measured ST (beta = 7.3, 95% CI 5.5 to 9.0, *p* < 0.001). Although EMA reports were highly related to accelerometer measures, the study showed that differences in the strength of association depend on various demographic characteristics and suggest that future research should use both EMA and accelerometers to measure activity to collect complementary activity data (Zink et al., 2018).

It is highlighted as strengths, the used an innovative method to identify SB in young adults. Although it is not a new method, it has only been more recently in the field of research on physical activity and health in the natural environment (Dunton, 2017).

In addition, the use of 7 days of collection using both methods (mEMA and accelerometry) may have provided more records with valid information (same moment) compared to the use of only 4 days, according to most studies of this nature (Marszalek et al., 2014). Another strong point was a larger number of participants with valid accelerometry data. Since it was not the purpose of the study to make a physical activity pattern over a traditional week, those who had at least one valid day of accelerometer use could be included in the analyses. Nevertheless, compared to other studies, this study advanced in a total number of participants and in the number of records. Another point that should be highlighted is the advance in the classification of presence or absence of SB, considering the counts/min value of each of the previous 5 min and not only the average of these counts in a specific period.

It is also important to highlight the use of criteria that enabled analyzing data in its concomitant period in time (mEMA and accelerometer). Some studies (Dunton et al., 2012; Liao et al., 2014; Bruening et al., 2016) did not consider EMA records when there was a window with more than 30 min of consecutive zeros (from the time of mEMA record).

Some limitations such as the occurrence of low return rates for the mEMA application (48.7%, ranging from 95.1 to 1.6%) also need to be highlighted. Furthermore, failure to use an objective method that discriminates the adoption of the participant’s position, such as an inclinometer, could have greatly aided in the classification of the presence/absence of the outcome (SB).

This study demonstrated the viability of using mEMA to obtain information about SB in different contexts and demonstrated good sensibility in the identification of the presence of this outcome in young adults. The concomitant use of these methods in future studies about SB in young adults is recommended.

## Author Contributions

CLPR designed the scientific work, drafted the first version of the manuscript, and gave the final approval of the manuscript. CLPR, CCLB, MBB, and GBS critically interpreted the data and gave the final approval of the manuscript. MR and ERVR supervised the scientific work, provided a critical revision of the work, and gave the final approval of the version to be published.

## Conflict of InterestStatement

The authors declare that the research was conducted in the absence of any commercial or financial relationships that could be construed as a potential conflict of interest.
